# Evaluation of complications of administration for tolvaptan after fluid resuscitation in critical care

**DOI:** 10.1186/2197-425X-3-S1-A545

**Published:** 2015-10-01

**Authors:** H Arimoto, H Rinka, T Miyaichi, A Fuke, J Ishikawa, T Morooka, K Shigemitsu, T Morimoto

**Affiliations:** Emergency and Critical Care Medical Center, Osaka City General Hospital, Osaka, Japan

## Background

Hypovolemic status (e.g. sepsis, trauma) is common situation in emergency department. The patients should be given a lot of fluid to prevent circulatory collapse with following early goal directed therapy or massive transfusion protocol in ED. ICU physician usually give some diuretics, as the patients are in hypervolemic status. Tolvaptan is an oral, non-peptide, selective antagonist of vasopressin V2-receptor whose action on the distal nephron causes loss of electrolyte-free water. We evaluate the side effects of tolvaptan for overhydrate status after fluid resuscitation in the critical care field.

## Methods

The indications of administration for tolvaptan are over-hydration and normal range of serum sodium and renal functions. 7.5mg or 15mg tolvaptan tablets were given once a day for a couple of day in refilling phase after fluid resuscitation. Electrolyte and blood gases were followed al least three times a day in all patients. The enrolled patients were divided into the normal-dose group and low-dose group. Any complications, especially serum sodium level, renal function and neurological outcome were compared.

## Results

A total of 62 patients were enrolled in this study. There was no significant difference in the both groups (the low-dose group; n = 29, average 8.6mg/day, the normal-dose group; n = 33, average 12.9mg/day). Though hypersodium was tend to occur in normal-dose group, any mortal complications were not seen in the both groups.

## Conclusions

Administration for tolvaptan is alternative method to improve over-hydration without serious adverse events. But strict collections of blood sample are recommended to prevent any complications such as central pontine myelinolysis.

